# Amyand's Hernia in a Neonate Presenting with Inguinoscrotal Erythema: A Difficult Diagnosis

**DOI:** 10.1055/s-0039-1697601

**Published:** 2019-10-31

**Authors:** Ahmed Mohamed, Anas Fagelnor

**Affiliations:** 1 Department of Paediatric Surgery, University Hospitals of Leicester NHS Trust, Leicester Royal Infirmary Infirmary Square Leicester, Leicester, United Kingdom of Great Britain and Northern Ireland; 2 Department of Pediatric Surgery, Benha Children Hospital, Benha, Egypt

**Keywords:** Amyand's, appendicitis, inguinal hernia, neonate

## Abstract

The presence of the vermiform appendix, whether inflamed or not, inside a hernial sac is known as Amyand's hernia. Due to nonspecific signs, preoperative diagnosis is not common and requires a high index of suspicion along with awareness of this rare entity. It is more commonly mistaken for a strangulated or incarcerated inguinal hernia. Most cases of reported Amyand's hernia with appendicitis were in pre-term babies, infants, and post-menopausal women. We present a similar case in a 19-day-old, full-term baby presenting with inguinoscrotal edema, erythema, and without a palpable inguinoscrotal mass.

## Introduction

Amyand's hernia is defined as an inguinal herniation of a normal, perforated, or acutely inflamed appendix. It was first described by Claudius Amyand in 1735, a French born surgeon working at St George's Hospital in London. On December 6, 1735, he performed the first recorded successful appendicectomy in an 11-year-old boy who had an inguinal hernia combined with an acutely inflamed appendix. 1 Amyand's hernia is a relatively rare presentation of appendicitis, with an incidence of less than 1% of all inguinal hernias according to the literature. 2 Although many presentations are described in the literature, the most common is one of an incarcerated or partially reducible inguinal hernia. We report a case of Amyand's hernia with appendicitis presenting only with inguinoscrotal edema and erythema in an almost 3-week-old baby.

## Case Report

**Fig. 1:**
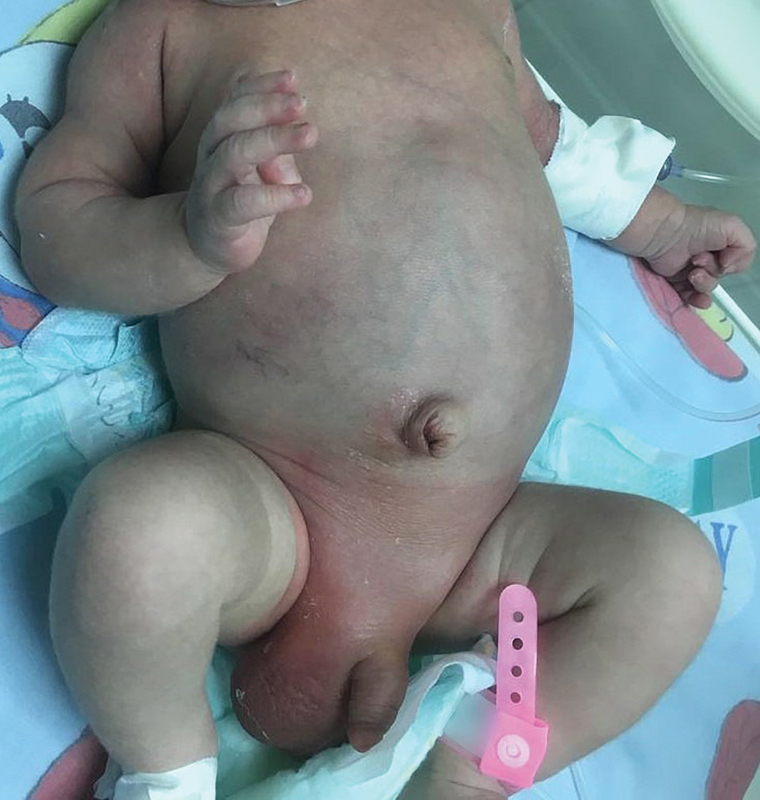
Neonate presenting with inguinoscrotal erythema and edema.

**Fig. 2:**
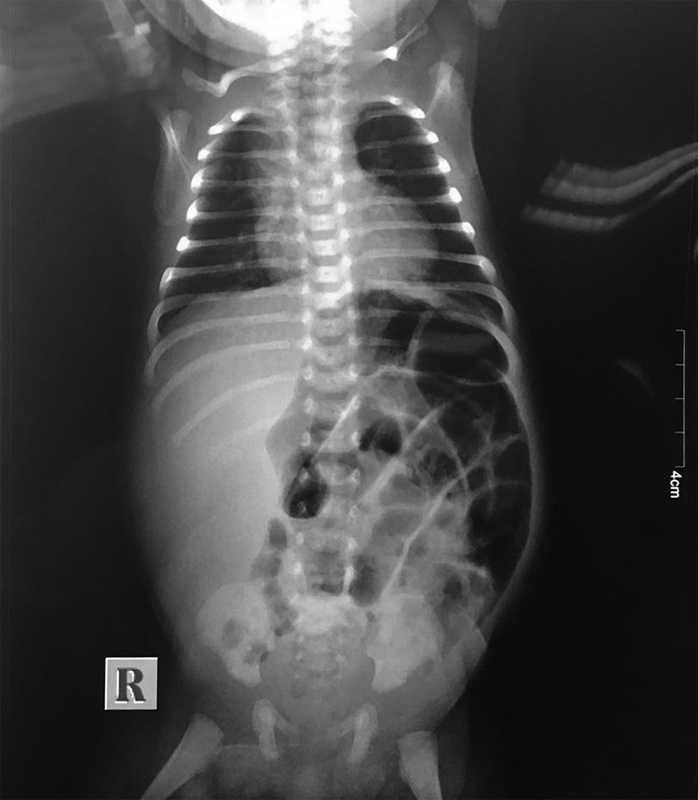
Preoperative abdominal X-ray.

Our patient is a 19-day-old, full-term baby, born in good general condition who presented to our emergency department with 1-day history of inguinoscrotal erythema and persistent irritability. The patient was struggling with achieving full feeds, although not vomiting and managing to open bowels normally. Examination revealed a distended, soft, nontender abdomen and bilateral scrotal edema along with moderate inguinoscrotal erythema and tenderness ( Fig. 1 ). Examination of the inguinal region revealed no evidence of irreducible hernia or inguinal thickening on either side. Laboratory investigations were normal apart from increased C-reactive protein levels. Due to the unclear signs, we resorted to imaging for answers. An abdominal X-ray showed some dilated bowel loops but no evidence of intestinal obstruction or aeration in the scrotal compartments ( Fig. 2 ). An inguinoscrotal ultrasound (US) with Doppler did not show any evidence of incarcerated bowel loops or testicular torsion, although pointing to increased vascularity and echogenicity of the right testis. The patient was, therefore, admitted on the working diagnosis of epididymo-orchitis and started on intravenous antibiotics.

**Fig. 3:**
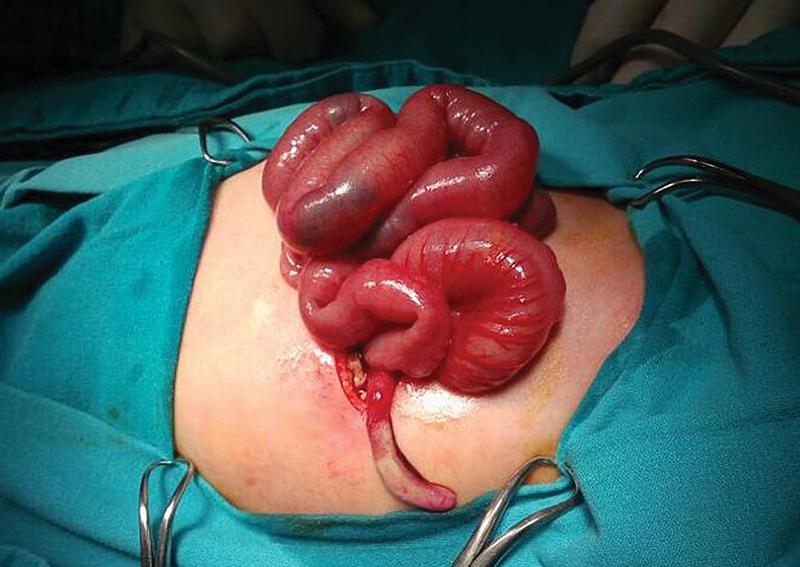
Intraoperative finding showing inflamed appendix.

Twelve hours later, our patient exhibited bilious vomiting and worsening inguinoscrotal edema. Abdominal examination showed signs of generalized rigidity and guarding. With no clinical evidence of an incarcerated hernia, the exclusion of testicular torsion by Doppler, and the development of an acute abdomen, we opted for an exploratory laparotomy through a right upper quadrant incision. This allowed us to be able to exclude any source of intra-abdominal sepsis while at the same time providing adequate surgical access to deal with the possibility of a missed irreducible hernia. The bowel was inspected and the appendix was found to be incarcerated in the right internal ring with the evidence of inflammation and suppuration ( Fig. 3 ). The appendix was gently reduced. Appendectomy and closure of the internal ring with a purse-string suture were done. Postoperatively, feeding was started through the nasogastric tube after 8 hours that was tolerated well and built up gradually. Antibiotics were discontinued in 5 days after improvement in inflammatory markers and resolution of the inguinoscrotal erythema. No other clinical evidence of associated comorbidities common with neonatal appendicitis was illustrated. The patient was discharged home and had an unremarkable 3-month follow-up.

## Discussion

Neonatal appendicitis is a rare condition associated with comorbidities such as prematurity or other diagnoses like Hirschsprung's disease, meconium plug syndrome, and cystic fibrosis. 3 Inflamed appendix within the hernial sac, on the other hand, accounts for one-third of the neonatal appendicitis described in the literature. Variable presentations ranging from irritability with signs of irreducible hernia to systemic signs of sepsis and shock. 4

Although the exact pathophysiology of this entity is unknown, one of the theories is the presence of a congenital band extending from the appendix to the right testis going through the inguinal canal. 5 A link between Amyand's hernia and appendicitis has also been debated. Some authors suggest that it is an incidental finding, while others arguing that the appendix becomes entrapped due to the contraction of abdominal muscles, which leads to compromised blood flow with consequent inflammation and bacterial overgrowth. 6

Most authors consider diagnosis of this condition to be intricate. This is because of the nonspecific manifestations, as in this case, where no obvious signs of irreducible hernia or intestinal obstruction were found. Cankorkmaz et al described a series of 12 cases, where only one was diagnosed preoperatively by US. All patients presented with an inguinal nonreducible mass and only a few exhibited fever, vomiting, or intestinal obstruction symptoms. These patients were taken to theater with a working diagnosis of incarcerated hernia. Intraoperatively they found six cases of Amyand's hernia with inflamed appendices: two normal appendices and four of equivocal nature. 7 Another larger case series described 46 patients diagnosed with Amyand's hernia, most of which after the incidental finding of the appendix in the hernial sac. 8 The differential diagnosis for a painful mass in the inguinal area may include incarcerated hernia, inguinal lymphadenitis, epididymo-orchitis, testicular torsion, or soft-tissue infection. Making the correct diagnosis preoperatively requires awareness of this rare entity by the clinician, as well as physical examination findings consistent with a hernia mass without obstructive signs. 6 Utilizing imaging like inguinoscrotal US was helpful in achieving a preoperative diagnosis in few reported cases. 9 In one series, US scan was successful in diagnosing 9 out of 12 patients by revealing a blind ending intestinal loop. 10 At the same time, Color Doppler US can be very useful in excluding testicular torsion from the differential diagnosis with an 80 to 98% sensitivity and an accuracy of 97%. 11 Moreover, computed tomography scan was able to visualize an inflamed appendix extending into the inguinal canal of a 77-year-old female. 2 Whether or not it is worthwhile exposing a neonate to such radiation to arrive at a diagnosis will be subject to controversy. Although no mortality is reported in the literature, delayed diagnosis and surgical intervention meant that on surgical exploration, many appendices were found to be perforated. Scrotal abscess, peritonitis, and a case progressing to septic shock are other reported complications. 4

Due to the majority of cases being incidentally found on surgical exploration, treatment is mainly by primary hernia repair and appendectomy through an inguinal incision. In cases with unclear diagnosis, reports of laparotomy as a primary incision, combined abdominal and inguinal incisions, and even scrotal explorations were described. 12 Some authors advocate against removing the appendix when found incidentally without any obvious signs of inflammation. 8 Laparoscopic approach is now gaining more popularity in such cases, as it allows increased visualization of the abdominal cavity and can be diagnostic and therapeutic. 12 However, utilizing this approach would be more likely if the patient's clinical condition is stable.

Diagnosing this rare entity requires a high index of suspicion by clinicians and when imaging is inconclusive, resorting to diagnostic laparoscopy should be considered to avoid laparotomy or multiple surgical incisions. While a tender, irreducible inguinal mass is the most common presentation, we report a case of Amyand's hernia with concurrent appendicitis presenting only with inguinoscrotal erythema and edema.

## Notes

New Insights & the Importance for the Pediatric Surgeon Diagnosing Amyand's hernia in neonates can be difficult. While a tender, irreducible inguinal mass is the most common presentation, we report a case of Amyand's hernia with concurrent appendicitis presenting only with inguinoscrotal erythema and edema.

## References

[1] Amyand C Of an inguinal rupture, with a pin in the appendix caeci incrusted with stone, and some observations on wound in the guts Philos Trans R Soc Lond B Biol Sci 1736 39 329 342

[2] Ash L Hatem S Ramirez G A Veniero J Amyand's hernia: a case report of prospective CT diagnosis in the emergency department Emerg Radiol 2005 11 04 231 232 16133611 10.1007/s10140-005-0411-6

[3] Jancelewicz T Kim G Miniati D Neonatal appendicitis: a new look at an old zebra J Pediatr Surg 2008 43 10 e1 e5 10.1016/j.jpedsurg.2008.05.014 18926195

[4] Shen Z Zheng S Timely recognition of Amyand's hernia with appendicitis in infants World J Pediatr 2015 11 04 392 394 24668237 10.1007/s12519-014-0474-0

[5] Baldassarre E Centonze A Mazzei A Rubino R Amyand's hernia in premature twins Hernia 2009 13 02 229 230 18791780 10.1007/s10029-008-0427-4

[6] Livaditi E Mavridis G Christopoulos-Geroulanos G Amyand's hernia in premature neonates: report of two cases Hernia 2007 11 06 547 549 17541492 10.1007/s10029-007-0242-3

[7] Cankorkmaz L Ozer H Guney C Atalar M H Arslan M S Koyluoglu G Amyand's hernia in the children: a single center experience Surgery 2010 147 01 140 143 19910011 10.1016/j.surg.2009.09.038

[8] Cigsar E B Karadag C A Dokucu A I Amyand's hernia: 11 years of experience J Pediatr Surg 2016 51 08 1327 1329 26707425 10.1016/j.jpedsurg.2015.11.010

[9] Sun X F Cao D B Zhang T Zhu Y Q Amyand's hernia in a neonate: a case report J Res Med Sci 2014 19 02 193 195 24778677 PMC3999609

[10] Okur M H Karaçay S Uygun I Topçu K Öztürk H Amyand's hernias in childhood (a report on 21 patients): a single-centre experience Pediatr Surg Int 2013 29 06 571 574 23417545 10.1007/s00383-013-3274-z

[11] Prando D Torsion of the spermatic cord: the main gray-scale and Doppler sonographic signs Abdom Imaging 2009 34 05 648 661 18709404 10.1007/s00261-008-9449-8

[12] Fascetti-Leon F Sherwood W Neonatal appendicitis and incarcerated inguinal hernia: case report and review of the literature J Indian Assoc Pediatr Surg 2017 22 04 248 250 28974880 10.4103/jiaps.JIAPS_226_16 PMC5615902

